# Editorial: Natural Killer Cells in Tissue Compartments

**DOI:** 10.3389/fimmu.2020.00258

**Published:** 2020-02-20

**Authors:** Massimo Vitale, Michael A. Caligiuri, Simona Sivori

**Affiliations:** ^1^UOC Immunologia, IRCCS Ospedale Policlinico San Martino, Genoa, Italy; ^2^Department of Hematology and Hematopoietic Cell Transplantation, City of Hope National Medical Center, Los Angeles, California, United States; ^3^Department of Experimental Medicine, University of Genoa, Genoa, Italy; ^4^Center of Excellence for Biomedical Research, University of Genoa, Genoa, Italy

**Keywords:** conventional NK cells, tissue resident NK cells, innate lymphoid cells, tissue microenvironment, NK receptors

Most of our current knowledge about human natural killer (NK) cells comes from studies on cells derived from peripheral blood, also known as “conventional” NK cells (c-NK), but recently, interest in the characterization of NK cells within tissues has increased, and besides recirculating cells, different tissue resident NK cells (tr-NK), each possessing distinct phenotypic profiles, have been described.

This Research Topic gathers the most recent information in the field to consolidate the emerging pictures of NK cells in the different organs, and to explain how the homeostasis of these unique NK cell subsets is normally maintained, or altered in pathologic conditions. The topic has successfully collected articles focused on a number of tissues, covering most of the compartments where NK cells are currently under study.

Two articles focus on the **bone marrow** (BM), where hematopoietic stem cells (HSC), or common lymphoid precursors (CLP) can generate mature NK cells or move to secondary lymphoid organs or peripheral tissues to differentiate under the influence of specific local microenvironments. By reviewing the recent literature and also their own data Bonanni et al. and Bozzano et al. depict a quite complex scenario in which BM, besides supporting NK cell and other innate lymphoid cell (ILC) development, can also orchestrate the NK cell mediated responses to infections. For example, a peculiar Lin^−^CD34^+^DNAM1^hi^CXCR4^+^ CLP subset with the potential of generating fully functional NK cells and reaching peripheral inflamed tissues can exit the BM upon prolonged systemic inflammation. Additionally, mature NK cells can also leave the BM, reach infected peripheral tissues and recirculate from the peripheral blood to the BM. Here, mature NK cells can undergo homeostatic or infection-induced proliferation contributing to their reservoir and also to the generation of “memory-like” long-lived NK cells. In T.Gondii-infected mice, BM NK cells can also induce, via IFNγ, regulatory monocytes to control exaggerated, tissue damaging, inflammatory responses. These NK cells could resemble the human BM-resident NK cell population characterized by low cytotoxicity and high IFNγ production.

The exit from BM of precursors or relatively immature NK cells emphasizes the question on the origins and homeostasis of specialized NK cell populations in specific tissues. This question applies, for example, to **the uterus**. Uterine NK cells (u-NK) represent a heterogeneous population endowed with peculiar functions, spanning from the support of embryo development, to the maternal-fetal tolerance, to the control of infection. Strikingly, this population undergoes important changes upon the transition from the steady state to pregnancy, e.g., u-NK cell frequency dramatically increases in the decidua after embryo implantation. How the dynamics of this population are regulated by the local proliferation of tr-NK cells and/or migration and adaptation of c-NK cells remains an interesting and incompletely addressed question. Based on data from murine virgin or pregnant uteri, Sojka et al. propose a two-wave hypothesis for u-NK cell accumulation during pregnancy. The first wave is due to the local proliferation of tr-NK cells during decidualization, whereas the second, occurring during the placentation, involves the recruitment of peripheral c-NK cells. Importantly, these c-NK cells participate in spiral arteriole remodeling by acting on endothelial and decidual stromal cells in an IFNγ-dependent way.

In healthy pregnancy, the pool of human decidual NKs includes poorly cytotoxic Tbet^pos^EOMES^pos^CD56^bright^CD16^−^KIR^+^ cells, expressing tissue residency markers (CD69, CD49a, integrin b7, and CD9), and even the inhibitory receptor, 2B4. However, these cells become fully active during viral infections, demonstrating their high plasticity. This issue is discussed by Jabrane-Ferrat, who suggests that the increased NK cell cytotoxicity depends on education via NKG2A- and/or KIR-mediated recognition of HLA molecules on fetal trophoblast cells, and on NKp46 signaling and/or cytokine stimulation during viral infections.

A suppressed u-NK cell phenotype and function may contribute to the progression of endometrial tumors. Degos et al. show that u-NK cells are minimally present in the tumor infiltrate, at least in part secondary to alterations in chemokines (CXCL12, IP-10, and CCL27) and cytokines (IL-1β and IL-6) that are present in the tumor microenvironment. Moreover, tumor resident CD103^+^ u-NK cells are characterized by reduced cytotoxicity and increased expression of inhibitory checkpoint receptors, such as TIGIT, and TIM-3, as compared to recruited CD103^−^ c-NK cells.

Three Research Topic articles focus on human **liver**, an organ in which NK cells represents almost 50% of all intrahepatic lymphocytes. As described in detail by Mikulak et al., human liver contains three NK cell populations showing transcriptional and phenotypic differences: liver tr-NK cells, memory-like NK cells and recirculating c-NK cells. Liver tr-NK cells are enriched in CD56^bright^ NK cells and are characterized by a peculiar transcription factor profile that includes Hobit, Tbet and increased Eomes expression. This profile is in line with the known liver tr-NK cell phenotype Eomes^hi^Tbet^*lo*^TIGIT^+^CD69^+^CXCR6^+^CD49e^−^. Retention of liver tr-NK cells is probably due to their expression of CXCR6, CXCR3, and CCR5. Indeed, these receptors can interact with CXCL16, CCL3, and CCL5 produced by cholangiocytes, liver sinusoidal endothelial cells, Kupffer cells and hepatocytes, thus favoring tr-NK cells homing to liver. According to the authors, these liver tr-CD56^bright^ NK cells also include an interesting small subset expressing CD49a and CD94:NKG2C that could be related to a clonal expansion in response to viral infections. These so–called memory-like tr-NK cells, however, may be different from the adaptive NK cells of the peripheral blood that develop/expand in CMV^+^ or CMV-reactivating donors. The situation is different for CD16^+^Siglec9^+^ NK cells found in the liver that are Tbet^+^Eomes^+^ and likely represent c-NK cells recirculating through the liver blood system without being retained in the organ.

Harmon et al. have analyzed the soluble factors produced in the liver microenvironment that can regulate the liver tr-NK cell phenotype in humans. In particular they analyze the role of TGF-β in suppression of Tbet and expression of Eomes in liver tr-NK cells. Notably, blocking TGF-β signaling through pre-treatment with a specific inhibitor reverses the phenotype of liver tr-NK cells toward that of peripheral blood c-NK cells. Interestingly, liver tr-NK cells share many phenotypic characteristics with u-NK cells (CD56^bright^ Eomes^hi^ CD69^+^), probably as a result of TFG-β in both the tissues.

The importance of the local microenvironment in shaping the NK cell compartment has also been demonstrated in non-alcoholic ratty liver diseases, namely in non-alcoholic steatohepatitis (NASH). In this study, Stiglund et al. highlight the phenotypic modification (i.e., an increased expression of NKG2D) of liver tr-NK cells as well as considerable alterations between liver and adipose tr-NK cells, as well as peripheral blood c-NK cells, independent of disease status.

Poggi et al. provide an overview of NK cells in the context of the **gut** lymphoid tissue. According to the authors' view, this scarce population, scattered within the lamina propria, can nevertheless play important roles in different pathologies, including inflammatory bowel diseases (IBD), and cancer. Indeed, although it is still uncertain as to the composition of this population in terms of c- or tr-NK cells, different studies referenced in the article suggest peculiar regulatory features of gut NK cells, which may influence TH1/TH17 or TH2 responses in IBD. On the other hand, the authors describe the anti-tumor function of gut NK cells in the context of certain subtypes of colon cancer.

An involvement of NK cells in pathogenic processes is highlighted in the review by Turner et al. on the **kidney**. NK cells represent ~25% of lymphocytes in healthy human kidney, and contain an important fraction of CD56^bright^ cells (37% of total kidney-NK cells). The expression of CD69, predominant in CD56^bright^ cells, and parabiosis experiments in mice, indicate that kidney harbors both tr- and c-NK cells. tr-NK cells are involved in both acute kidney injury, being attracted, and activated by damaged tubular epithelial cells, and in chronic kidney disease, supporting the progression of interstitial fibrosis. c-NK cells may be of pathogenic relevance during kidney graft rejection, indeed they can induce ADCC against the allograft by the CD16-mediated binding of donor-specific antibodies.

Two Research Topic articles focus on **lung** NK cells. By reviewing different manuscripts in the field, Hervier et al. describe the landscape of NK cells present in the lung. The majority (up to 80%) of these cells displays a mature CD56^dim^CD16+ phenotype and circulate between the organ and peripheral blood. The remaining cells include the lung tr-NK cells that are characterized by the CD16-CD49a+CD69+CD103+ surface phenotype. Also, in this case, the impact of the local microenvironment likely plays a role in shaping the phenotypic and functional features of lung tr-NK cells. Interestingly, Scharenberg et al. describe marked NK cell hyperresponsiveness, particularly in CD56^bright^CD16- NK cells, during Influenza A virus infection, both in human lung and blood.

Bonaccorsi et al. focus on a still poorly investigated issue: the possible role of NK cells in the **carotid atherosclerotic plaques (CAP)** formation. They show that NK cells are present in the plaques, primarily in symptomatic patients, and are enriched for CD56^bright^ perforin^low/neg^ cells and express markers of tissue residency (i.e., CD103, CD69, and CD49a). Such plaque tr-NK cells (CAP-NK) might preferentially be recruited via CCR7 (as high levels of CCL19 and CCL21 could be measured in the plaque) and then induced to up-regulate markers of tissue residency under the influence of local inflammation. According to the authors, CAP-NK cells may participate in the disease process by favoring both the progression of the atherosclerotic process within carotid plaques and promoting plaque instability. Indeed, CAP-NK cells produce the pro-inflammatory cytokine IFNγ, which can also induce matrix metalloproteinases (MMPs) possibly affecting plaque stability. On the other hand, MMPs may also contribute to the abundant shedding of activating NK-receptor ligands found in the plaques.

An additional tissue to be considered when studying NK cells is that of the tumor. Although this issue could be the subject of a dedicated Research Topic, we here present a few contributions highlighting two specific components when considering any NK cell-based immunotherapeutic strategy: NK cell checkpoints and tumor infiltration by NK cells.

Pesce et al. analyze **peritoneal carcinomatosis (PC)** and describe a compromised function of NK cells present in the peritoneal fluid (PF) of high-grade, and even low-grade, PC patients. PF NK cells of low-grade PC patients include a large fraction of immature NKG2A+KIR-CD57-CD16^dim^ cells characterized by a strong downregulation of the main activating NK cell receptors (such as NKp30, DNAM-1, and CD16). By contrast, and perhaps interestingly, PF NK cells of high-grade PC patients have a mature phenotype (KIR+ CD57+ NK cells) but show increased expression of the immune checkpoint PD-1.

Parodi et al. analyze NK cells in setting of tissue **hypoxia**, a condition frequently occurring within the tumor itself. Besides presenting the hypoxia-induced transcriptome of NK cells, the study also highlights how the decreased O_2_ tension can influence the pattern of chemokine receptor expression, ultimately favoring the migration of poorly cytotoxic CD56^bright^CD16^dim^ NK cells. Thus, local conditions, such as hypoxia, can directly act on the expression of specific chemokine receptors and affect migration of specific NK cell subsets, thereby having a significant impact on tumor escape from effective immune surveillance.

In this context, Ingegnere et al. have described a new (virus-free) protocol for the production of NK cells transfected with the CCR7 chemokine receptor. Interestingly, this transfection method could be applied to other chemokine receptors to induce NK cell migration to different tissues and tumor sites. This approach may be combined with the transfection of NK cells with chimeric antigen receptors (CAR) that target tumor-associated antigens (i.e., **CAR-NK cells**).

Although not initially considered in the original framework of this issue on tissue NK cells, we decided to invite some additional contributions focused on **ILCs**, due to the now obvious relationship between NK cell differentiation and other ILC subsets (namely group 1 ILCs), as well as their reciprocal functional interactions. A Review article by Mariotti et al. discusses the topical issue of the checkpoint receptors, their expression on NK cells and other ILCs, and the known and hypothesized implications in cancer, in defense against infectious pathogens, and in pregnancy. Two research articles by Manson et al. and Carvelli et al. focus on trauma and hemorrhagic or septic shock, respectively, and on the related altered immune responses which often result in adverse outcomes. In particular, the changes within ILCs or with innate-like lymphocytes are investigated and the possible consequences on immune homeostasis are discussed.

Finally, a collection of articles on tissue NK cells and ILCs could not be complete without consideration of their common key receptors, the natural cytotoxicity receptors (**NCRs**). Two reviews, by Barrow et al. and Parodi et al., focus on this issue, providing a thorough and updated picture of these receptors in both NK cells and ILCs. Interestingly, both the articles highlight the polyfunctionality of these receptors, consistent with the well-established multifaceted role of NK cells and ILCs in different tissue compartments.

## Author Contributions

All authors listed have made a substantial, direct and intellectual contribution to the work, and approved it for publication.

### Conflict of Interest

The authors declare that the research was conducted in the absence of any commercial or financial relationships that could be construed as a potential conflict of interest.

